# Excessive polypharmacy and potentially inappropriate prescribing in 147 care homes: a cross-sectional study

**DOI:** 10.3399/BJGPO.2021.0167

**Published:** 2021-11-24

**Authors:** Clare MacRae, David AG Henderson, Stewart W Mercer, Jenni Burton, Nicosha De Souza, Paula Grill, Charis Marwick, Bruce Guthrie

**Affiliations:** Centre for Population Health Sciences, Usher Institute, University of Edinburgh, Edinburgh, UK 1; 2 Institute of Cardiovascular and Medical Sciences, University of Glasgow, Glasgow, UK; 3 Population Health and Genomics, School of Medicine, University of Dundee, Dundee, UK

**Keywords:** general practice, polypharmacy, prescribing safety, care homes, older people, drug-related side effects and adverse reactions

## Abstract

**Background:**

Care home residents often have multiple cognitive and physical impairments, and are at high risk of adverse drug events (ADEs).

**Aim:**

To describe excessive polypharmacy and potentially inappropriate prescribing predisposing care home residents to ADEs.

**Design & setting:**

A cross-sectional analysis of all dispensed prescriptions for 147 care home residents in Tayside and Fife, Scotland.

**Method:**

Prevalence of excessive polypharmacy was examined using multilevel logistic regression, by modelling associations between individual and care home predictors with excessive polypharmacy (≥10 drugs). Prescribing of drugs known to increase the risk of eight clinically important ADE categories was examined. Drugs prescribed within each ADE category, for each resident, were counted.

**Results:**

In total, 32.3% (*n* = 1444/4468) of residents had excessive polypharmacy, which was more common in residents aged 70–74 years (adjusted odds ratio [aOR] 1.86, 95% confidence interval [CI] = 1.04 to 3.34) and 80–84 years (aOR 1.75, 95% CI = 1.01 to 3.02), living in a residential care home (aOR 1.50, 95% CI = 1.19 to 1.88), and located in Fife (aOR 1.37, 95% CI = 1.09 to 1.71). Excessive polypharmacy was less common in residents with dementia (aOR 0.73, 95% CI = 0.64 to 0.84), and 8.9% (95% CI = 5.9% to 11.6%) of the variation was attributable to care home predictors. Potentially inappropriate prescribing of ≥2 drugs was seen across all ADE categories, with highest prevalence seen in drugs predisposing to constipation (35.8%), sedation (27.7%), and renal injury (18.0%).

**Conclusion:**

Excessive polypharmacy is common in care home residents and is associated with both individual and care home predictors. Potentially inappropriate prescribing of drugs that predisposed residents to all included ADE categories is common. Research is needed to support and evaluate safe care home prescribing practices.

## How this fits in

Care home residents often have complex care needs owing to multiple cognitive and physical impairments that put them at increased risk of ADEs. Several studies refer to polypharmacy within care home residents, but few have examined prescribing systematically according to body system, associations between individual and care home predictors and excessive polypharmacy, or potentially inappropriate prescribing patterns predisposing to specific ADEs. This study finds that excessive polypharmacy and potentially inappropriate prescribing of drugs that increase risk of ADEs is common in this population. Research is needed to support and evaluate care home prescribing practices to achieve the optimum balance between symptomatic relief of symptoms and provision of long-term preventive therapies for this vulnerable population.

## Introduction

Care home residents are often older adults who are frail, with complex care needs owing to multiple cognitive and physical impairments.^1^ However, the majority of clinical guidance used by clinicians for this population is underpinned by clinical trials that usually exclude older adults who are frail,^2^ therefore advocating silo medicine through optimising single disease treatment.^3^ Application of these clinical guidelines, by multiple prescribers, from different specialties, in different locations, can result in a cascade of prescribing over a lifetime that results in polypharmacy, some of which may be owing to the treatment of avoidable ADEs with more medicines.^4^ Older adults are vulnerable to ADEs owing to age and disease-related changes in renal, cognitive, and sensory function, and altered pharmacokinetics and pharmacodynamics,^5^ making it difficult to distinguish between disease-related symptoms and ADEs.^6^ Residents are therefore at risk of polypharmacy and potentially harmful, often preventable, drug–drug and drug–disease interactions.^3,7^

Despite its importance, there is no consensus on the definition of polypharmacy. Count definitions are common where prescribing of ≥5 and ≥10 different drugs define polypharmacy and excessive polypharmacy, respectively.^7,8^ Other definitions focus on the appropriateness of polypharmacy (where all drugs are prescribed for the purpose of achieving specific therapeutic objectives and therapy has been optimised to reduce the risk of ADEs), irrespective of count. Polypharmacy is likely inappropriate when ≥1 drugs are prescribed that were never strongly indicated, where the indication has expired, or where one or a combination of drugs put the person at an unacceptably high risk of an ADE.

ADEs are estimated to be the primary cause of one in ten hospital admissions in older adults,^9^ often relating to ADEs such as cognitive impairment, falls,^10^ and renal injury.^11^ There is positive correlation between the number of ADE risk-increasing drugs and risk of developing an ADE; for example, co-prescription of psychotropic and cardiovascular medications that predispose to falls.^10^ Appropriate use of medications can increase longevity, reduce hospital admissions, and improve quality of life. However, the inappropriate polypharmacy is associated with increased drug costs, use of healthcare services, and symptoms that reduce a person’s quality of life.^12^ The aim of this study was to examine the prevalence of excessive polypharmacy and potentially inappropriate prescription of drugs that increase risk of ADEs, within a large nationally representative cohort of care home residents.

## Method

A cross-sectional analysis of prescribing was performed in all residents aged ≥60 years in care homes for older adults in two UK NHS health board areas, Tayside and Fife. The NHS provides universal healthcare coverage for all residents and no fees are required in payment for prescribing of medications. ‘Care home’ is an umbrella term for long-term care settings in the UK. Both residential and nursing care homes provide 24-hour care and support for adults with a range of needs.

Residents were identified by matching each individual’s residential address to Care Inspectorate care home registered addresses,^13^ and characteristics determined from publicly available Care Inspectorate data.^14^ Prescribing records, including all dispensed medications from community pharmacies within the study area, were linked with demographic data. Anonymised data were provided by the University of Dundee Health Informatics Centre^15^ and held in the ISO270001 and NHS Scotland accredited safe haven.

Excessive polypharmacy was defined as prescription of ≥10 distinct drug classes determined by subsections of the *British National Formulary (BNF)*,^16^ expanded as necessary to ensure that drug classes contained drugs with distinct mechanisms of action.^7^ Distinct drugs that are frequently co-prescribed (for example, subsection 2.9 antiplatelet drugs) were expanded. Drugs within combination preparations were counted separately (for example, co-codamol as paracetamol and codeine). Prescriptions for medical appliances (for example, stoma preparations and glucose-testing strips), were excluded, avoiding overcounting of polypharmacy. 'Current' prescribing was defined as any prescription in the 56 days before 31 March 2017. Repeat prescriptions for care home residents in these regions are frequently issued in 28-day cycles. Therefore, inclusion of two prescribing cycles was used to optimise capture of repeat prescribing, as well as highly interacting drugs, which are not always given daily or issued every 28 days (for example, sleeping tablets or analgesics). Individual predictors were age, sex, and dementia status, which was defined by any prior record of dementia during a hospital admission or any prior prescription for a dementia drug (*BNF* 4.11). Care home predictors were care home status (nursing or residential), size (small, medium, or large), Risk Assessment Document (RAD) score (a risk-assessment score categorised as low, medium, and high risk; high-risk score correlating with prioritised inspections),^17^ and NHS health board (Tayside or Fife). Deprivation status (Scottish Index of Multiple Deprivation [SIMD]) was not analysed because postcode assigned SIMD is a poor marker of individual socioeconomic status in this population as care home residents frequently migrate to new areas for care home placement.

Associations between individual and care home predictors with excessive polypharmacy were examined through multilevel logistic regression with random intercept, accounting statistically for clustering within care homes. Multicollinearity was assessed using χ^2^ hypothesis tests between categorical variables and generalised variation inflation factor for higher order correlations. Variance was partitioned in an empty model to estimate the intraclass correlation coefficient (ICC). Final independent predictor selection for the adjusted model was guided by minimisation of the Akaike Information Criterion and included predictors with significant univariate odds ratios (ORs). Potentially inappropriate prescribing of drugs known to increase ADEs risk associated with adverse clinical outcomes and/or reduced quality of life in older people was measured. ADE categories included anticholinergic effects, bleeding, constipation, heart failure, hypotension, renal injury, sedation, and urinary retention. The number of drugs prescribed within each ADE category for each resident was counted, then cross-tabulated by age according to drug lists developed through reference to Scottish Government Polypharmacy Guidance,^18^ drug summary of product characteristics (SPC),^19^ and the *BNF* ^16^ (see Supplementary Appendix S1). Anticholinergic drugs with modified Anticholinergic Risk Score (mARS) of ≥2 were included.^20^ aORs were reported with 95% CIs and statistical significance of 5%. All statistical analyses and plotting was undertaken using R (version 3.2.5).

## Results

### Demographics

A total of 4468 people aged ≥60 years in NHS Tayside and Fife regions were resident in 147 care homes for older people on 31 March 2017 (Table 1). Mean age of residents was 84.9 years (standard deviation [SD] 8.1), 3196 (71.5%) were female, and 2160 (48.3%) had a diagnosis of dementia. There were 2601 (58.2%) residents living in 71 care homes providing nursing care, while 3749 (83.9%) of residents lived in 117 privately owned care homes. Similar numbers of residents lived in medium and large, rather than small, care homes (2028 [45.4%], 1800 [40.3%], and 637 [14.3%], respectively), and the majority lived in care homes with a low-risk RAD score (3039 [68.0%]). Most residents lived in 90 care homes located in NHS Tayside (2676 [59.9%]).

**Table 1.** Resident and care home characteristics

Characteristic	Category	**Dispensed 0–9 drugs,** *n* = 3024, *n* (%)^a^	**Dispensed** ≥**10 drugs,** *n* = 1444, *n* (%)^a^	**All residents,** *n* = 4468, *n* (%)^a^
Sex	Male	850 (28.1)	422 (29.2)	1272 (28.5)
	Female	2174 (71.9)	1022 (70.8)	3196 (71.5)
Age, years	Mean (SD)	85.1 (8.3)	84.6 (7.9)	84.9 (8.1)
Age group, years	60–64	68 (2.2)	19 (1.3)	87 (1.9)
	65–69	101 (3.3)	53 (3.7)	154 (3.4)
	70–74	184 (6.1)	101 (7.0)	285 (6.4)
	75–79	317 (10.5)	163 (11.3)	480 (10.7)
	80–84	580 (19.2)	302 (20.9)	882 (19.7)
	85–89	765 (25.3)	393 (27.2)	1158 (25.9)
	90–94	709 (23.4)	288 (19.9)	997 (22.3)
	≥95	300 (9.9)	125 (8.7)	425 (9.5)
Drugs prescribed^b^	Mean (SD)	—	—	7.8 (4.3)
	Median (IQR)	—	—	8 (3–13)
Number of *BNF* chapters drugs prescribed from	Mean (SD)	3.4 (1.5)	6.1 (1.3)	4.2 (1.9)
Dementia diagnosis	No dementia	1506 (49.8)	802 (55.5)	2308 (51.7)
	Dementia	1518 (50.2)	642 (44.5)	2160 (48.3)
Care home type	Nursing care (*n* = 71 homes)	1858 (61.4)	743 (51.5)	2601 (58.2)
	Residential care (*n* = 76 homes)	1166 (38.6)	701 (48.5)	1867 (41.8)
Service sector	Private (*n* = 117)	2625 (86.8)	1124 (77.8)	3749 (83.9)
	Local Authority (*n* = 19)	246 (8.1)	205 (14.2)	451 (10.1)
	Third sector (*n* = 11)	153 (5.1)	115 (8.0)	268 (6.0)
Care home size^c^	Small (*n* = 41)	417 (13.8)	220 (15.2)	637 (14.3)
	Medium (*n* = 68)	1314 (43.5)	714 (49.4)	2028 (45.4)
	Large (*n* = 37)	1290 (42.7)	510 (35.3)	1800 (40.3)
RAD score	Low (*n* = 102)	1994 (65.9)	1045 (72.4)	3039 (68.0)
	Medium (*n* = 19)	421 (13.9)	175 (12.1)	596 (13.3)
	High (*n* = 26)	609 (20.1)	224 (15.5)	833 (18.6)
Health board	Tayside (*n* = 90)	1871 (61.9)	805 (55.7)	2676 (59.9)
	Fife (*n* = 57)	1153 (38.1)	639 (44.3)	1792 (40.1)

^a^Unless otherwise stated. ^b^Distribution normal overall but skewed in groups so SD and IRQ not reported for groups. Overall distribution shown in Supplementary Figure S1. ^c^Missing data for one care home. *BNF* = *British National Formulary*. IQR = interquartile range. RAD = Risk Assessment Document. SD = standard deviation.

### Prescribing

The mean and median number of individual drugs dispensed per person was 7.8 (interquartile range [IQR] 3.5–12.1) and 8 (IQR 3–13), respectively, for all residents (Table 1, Supplementary Figure S1).

All residents were prescribed drugs from mean 4.2 (SD 1.9) *BNF* chapters, and residents with excessive polypharmacy were prescribed drugs from mean 6.1 (SD 1.3). *BNF* chapters with highest prevalence of prescribing were: 4 — Central Nervous System, 1 — Gastrointestinal System, and 2 — Cardiovascular System, with 82.5%, 66.9%, and 64.5% of residents receiving ≥1 drug prescriptions, respectively (Table 2, Supplementary Figure S2). Chapters 9 — Nutrition and Blood (45.0%), 13 — Skin (39.1%), and 6 — Endocrine System (37.1%) also had high frequency prescribing of ≥1 drug. Chapters with lowest frequency of prescribing were: 7 — Obstetrics, Gynaecology and Urinary-Tract Disorders (9.0%), 12 — Ear, Nose and Oropharynx (3.0%), and 8 — Malignant Disease and Immunosuppression (1.9%).

**Table 2.** Number of care home residents, mean number of medicines, and most frequent drug class prescribed, by *BNF* chapter

		Most frequent drug class prescribed
***BNF* chapter**	Patients prescribed medicine, *n* (%)	Medications prescribed, mean (SD)	First (%)	Second (%)	Third (%)	Fourth (%)	Fifth (%)
1. Gastrointestinal System	2991 (66.9)	1.63 (0.84)	Osmotic laxatives (36.3)	Proton pump inhibitors(35.8)	Stimulant laxatives (17.7)	H_2_ receptor agonists (5.2)	Compound alginates and proprietary antacids (3.9)
2. Cardiovascular System	2883 (64.5)	2.32 (1.3)	Statins (27.3)	Loop diuretics (20.6)	Aspirin (17.8)	Beta-adrenoceptor blocking drugs (17.5)	Clopidogrel (14.4)
3. Respiratory System	684 (15.3)	1.79 (1.15)	Short acting beta_2_ adrenoceptor agonists (6.1)	Inhaled corticosteroids (5.6)	Non-sedating antihistamines (4.9)	Antimuscarinic bronchodilators (3.3)	Sedating antihistamines (1.7)
4. Central Nervous System	3687 (82.5)	2.39 (1.37)	Paracetamol (47.2)	Drugs for dementia (26.4)	SSRI antidepressants (19.0)	Other antidepressants (16.7)	Opioid analgesics (16.3)
5. Infections	1402 (31.4)	1.41 (0.74)	Broad spectrum penicillins (11.4)	Sulfonamides and trimethoprim (8.1)	Nitrofurantoin (6.2)	Penicillinase-resistant penicillins (4.2)	Tetracyclines (4.1)
6. Endocrine System	1658 (37.1)	1.26 (0.56)	Thyroid hormones (19.2)	Bisphosphonates and drugs affecting bone metabolism (10.1)	Biguanides (5.3)	Treatment corticosteroids (3.4)	Male sex hormones and antagonists (3.3)
7. Obstetrics, Gynaecology and Urinary-Tract Disorders	404 (9.0)	1.07 (0.27)	Drugs for urinary frequency, enuresis, incontinence (5.3)	Drugs for urinary retention (3.4)	Vaginal and vulval infections (0.5)	Topical hormone replacement therapy(0.4)	Drugs used in urological pain (0.1)
8. Malignant Disease and Immunosuppression	83 (1.9)	1 (0)	Hormone antagonists (1.5)	Azathioprine (0.2)	Other antineoplastic (0.1)	Mycophenolate (<0.1)	Ciclosporin (<0.1)
9. Nutrition and Blood	2011 (45.0)	1.98 (0.78)	Vitamin D (35.6)	Oral iron (10.2)	Drugs used in megaloblastic anaemias (9.9)	Fluoride (1.1)	Calcium supplements (0.5)
10. Musculoskeletal and Joint Diseases	692 (15.5)	1.09 (0.31)	Rubefacients, topical NSAIDs, capsaicin, and poultices (11.7)	Gout and cytotoxic induced hyperuricaemia (2.4)	Skeletal muscle relaxants(1.5)	Methotrexate (0.4)	Nonselective NSAIDs (0.4)
11. Eye	595 (13.3)	1.46 (0.82)	Tear deficiency, ocular lubricants and astringents (7.6)	Glaucoma: prostaglandinanalogues (3.8)	Antibacterial eye preparations (2.2)	Glaucoma: other (1.4)	Glaucoma: beta-blockers (0.9)
12. Ear, Nose and Oropharynx	133 (3.0)	1.06 (0.24)	Nasal allergy, topical antihistamines and cromoglicate (1.6)	Drugs for oral ulceration and inflammation (0.5)	Otitis externa (0.3)	Oropharyngeal anti-infective drugs (0.3)	Topical nasal decongestants (0.2)
13. Skin	1747 (39.1)	1.65 (0.97)	Emollients (23.1)	Barrier preparations (15.8)	Topical corticosteroids (9.0)	Antifungal skin preparations (2.6)	Shampoos and other preparations for scalp and/or hair (1.7)

*BNF* = *British National Formulary*. SD = standard deviation. SSRI = selective-serotonin reuptake inhibitors. NSAIDs = non-steroidal anti-inflammatory drugs.

The most prescribed drug classes were paracetamol (47.2%), osmotic laxatives (including lactulose and macrogols) (36.3%), and proton pump inhibitors (35.8%) (Table 2). Statins (27.3%), drugs for dementia (26.4%), emollients (23.1%), and loop diuretics (20.6%) were prescribed in over one-fifth of residents.

### Excessive polypharmacy

Excessive polypharmacy was present in 1444 (32.3%) residents, with the distribution of males and females similar to the overall population (Table 1). Calculation of the ICC in a null model showed that a large amount of the variation in excessive polypharmacy was owing to differences between care homes (8.9%, 95% CI = 5.9% to 11.6%) (Table 3).

**Table 3.** Multilevel logistic regression analysis of excessive polypharmacy in care home residents

Characteristic	Category	**Dispensed ≥10 drugs,** *n* = 1444, *n* (%)	**OR univariable**(**95% CI**)^b,c^	**OR multivariable**(**95% CI**)^c,d^
Age group, years	60–64	19 (21.8)	Ref	Ref
	65–69	53 (34.4)	1.76 (0.94 to 3.29)	1.80 (0.96 to 3.38)
	70–74	101 (35.4)	1.79 (1.00 to 3.21)	1.86 (1.04 to 3.34)
	75–79	163 (34.0)	1.67 (0.96 to 2.94)	1.73 (0.98 to 3.03)
	80–84	302 (34.2)	1.65 (0.96 to 2.85)	1.75 (1.01 to 3.02)
	85–89	393 (33.9)	1.60 (0.93 to 2.75)	1.66 (0.97 to 2.87)
	90–94	288 (28.9)	1.18 (0.68 to 2.04)	1.19 (0.69 to 2.07)
	≥95	125 (29.4)	1.14 (0.64 to 2.01)	1.08 (0.61 to 1.92)
Sex	Male	422 (33.2)	Ref	–
	Female	1022 (32.0)	0.89 (0.77 to 1.03)	–
Dementia status	No dementia	802 (34.7)	Ref	Ref
	Dementia	642 (29.7)	0.78 (0.68 to 0.89)	0.73 (0.64 to 0.84)
Care home type	Nursing	743 (28.6)	Ref	Ref
	Residential	701 (37.5)	1.49 (1.19 to 1.85)	1.50 (1.19 to 1.88)
RAD score	Low	1045 (34.4)	Ref	Ref
	Medium	175 (29.4)	0.75 (0.54 to 1.06)	0.72 (0.52 to 0.99)
	High	224 (26.9)	0.69 (0.51 to 0.93)	0.77 (0.56 to 1.04)
NHS health board	Tayside	805 (30.1)	Ref	Ref
	Fife	639 (35.7)	1.24 (0.99 to 1.57)	1.37 (1.09 to 1.71)
Intraclass correlation coefficient, % (95% CI)	—	—	8.9 (5.9 to 11.6)Null model	7.1 (4.2 to 9.5)Adjusted model

^a^Within category %. ^b^Number of groups = 147; residents in all models = 4468. Service sector and care home size not fitted in models as collinear with care home status. ^c^Unless otherwise stated. ^d^C-statistic = 0.685. – = variable not fitted. RAD = Risk Assessment Document.

**Figure 1.**
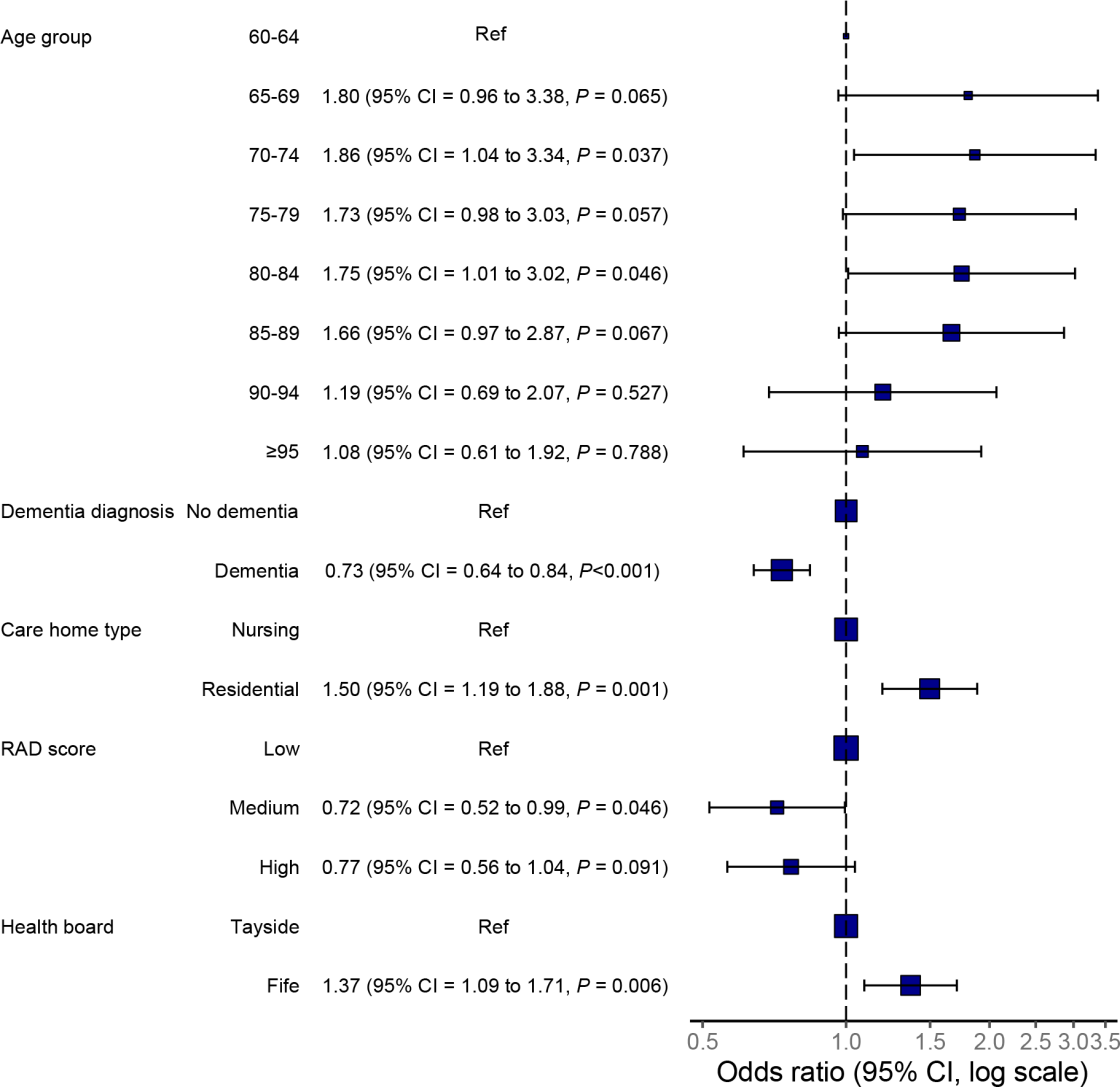
Multilevel logistic regression analysis of excessive polypharmacy in care home residents, adjusted model. RAD = Risk Assessment Document.

In the adjusted multilevel model, excessive polypharmacy was more common in residents aged 70–74 years (aOR 1.86, 95% CI = 1.04 to 3.34) and 80–84 years (aOR 1.75, 95% CI = 1.01 to 3.02), compared with residents aged 60–64 years (Figure 1). Residents living in residential care homes were more likely to have excessive polypharmacy than those in nursing care homes (aOR 1.50, 95% CI = 1.19 to 1.88), as were residents living in the NHS Fife area (aOR 1.37, 95% CI = 1.09 to 1.71) (Table 3, Figure 1). Residents with dementia (aOR 0.73, 95% CI = 0.64 to 0.84) and those living in care homes with medium RAD score (aOR 0.72, 95% CI = 0.52 to 0.99 versus care homes with low RAD score) were less likely to have excessive polypharmacy. No statistically significant differences were found between females and males.

### Prescribing of drugs associated with ADEs

**Figure 2.**
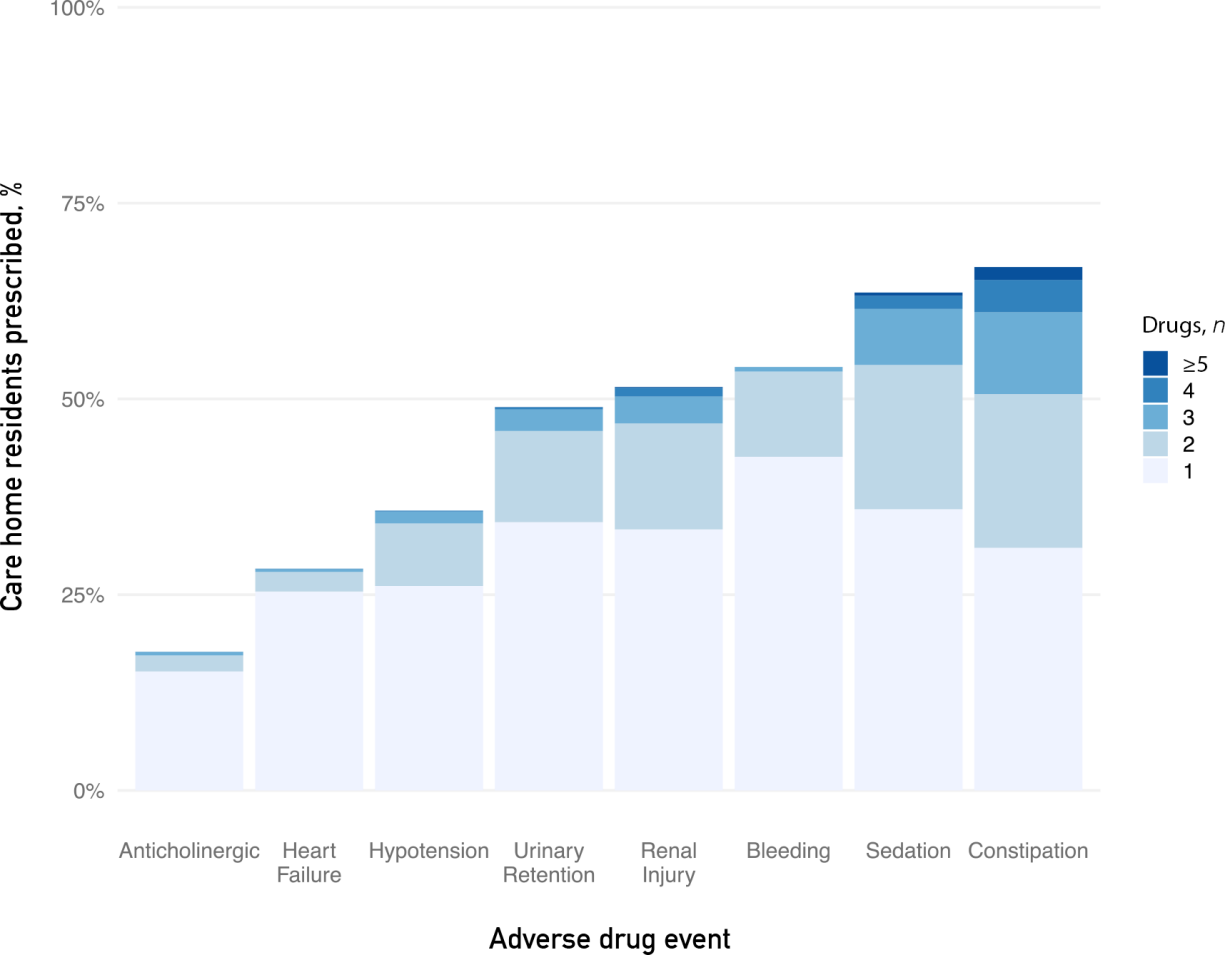
Percentage of care home residents prescribed drugs associated with eight common adverse drug events (ADEs)

Potentially inappropriate prescribing of drugs within all ADE categories was common, ranging from 17.7% (anticholinergic) to 66.8% (constipation) of residents prescribed ≥1 drug (Figure 2, Supplementary Table S1). Prescribing of any drugs predisposing to sedation, bleeding, and renal injury was found in 63.6%, 54.0%, and 51.5% residents, respectively. Categories with highest levels of prescribing of ≥2 drugs were constipation (35.8%), sedation (27.7%), and renal injury (18.0%). Co-prescribing of the highest number of drugs within the same category, ≥4 drugs, was seen in constipation (5.8%) and sedation (2.1%).

Older residents had more prescribing predisposing to renal injury and hypotension, and less predisposing to sedation and urinary retention (see Supplementary Table S2 and Supplementary Figure S3). Drugs predisposing to anticholinergic effects, heart failure, bleeding, and constipation did not show variation by age.

The most prescribed drug classes within the constipation category were loop diuretics (21.1%), opiates (20.5%), and beta-blockers (18.0%) (see Supplementary Table S3). Prescribing of drug classes predisposing to sedation included opiates (20.5%), selective-serotonin reuptake inhibitors (SSRIs) (19.5%), and beta-blockers (18.0%). The most prescribed drug classes predisposing to renal injury included loop diuretics (21.1%), penicillin antibiotics (11.3%), and angiotensin-converting enzyme inhibitors (10.6%).

## Discussion

### Summary

The mean and median number of individual drugs dispensed per person was 7.8 (SD 4.3) and 8 (IQR 3–13), respectively. Care home predictors accounted for 8.9% (95% CI = 5.9% to 11.6%) of variation in rates of excessive polypharmacy. Excessive polypharmacy was more likely in residents living in residential care homes, and those living in NHS Fife.

Highest prescribing rates were seen for drugs relating to the central nervous, gastrointestinal, and cardiovascular systems. Prescribing ≥1 drug known to increase the risk of the ADE categories ranged from 17.7% (anticholinergic) to 66.8% (constipation). Prescribing of ≥2 drugs ranged from 2.4% (anticholinergic) to 35.8% (constipation). Drugs used to manage cardiovascular disease, pain, and mental health conditions were most prescribed within the ADE categories.

### Strengths and limitations

The strengths of the study include the comprehensive assessment of all dispensed medications for the entire population of care home residents in two health board regions, systematically examining prescribing according to body system, and using multilevel analysis to account for clustering of residents within many care homes. Prescriptions for medical appliances were excluded, therefore avoiding overcounting of polypharmacy. ADE category drug lists were formulated with reference to validated sources.^16,19^ Current prescribing was defined as all drugs issued in the last 56 days, therefore including recent short courses of medications, which is important as such drugs often have important interactions and/or commonly cause adverse events; for example, antibiotics and analgesics.

The study has several limitations, including the lack of clinical diagnoses other than dementia status, meaning that clinical appropriateness cannot be evaluated (although ‘indication’ in the very frail is not straightforward given the lack of evidence in this population). Clinical diagnoses, derived measures of multimorbidity and/or frailty, and GP prescribing practices were not available and would be valuable factors to include in further research. In addition, 48.3% of residents were identified by routine data as having dementia. This figure is lower than the previous estimate of 62% of Scottish long-stay care home residents, likely reflecting that dementia in this population is not always coded.^21^ Additional clinical detail could support application of more formal tools, such as the STOPP/START^22^ or Beers Criteria,^23^ for potentially inappropriate medication use in older adults.

### Comparison with existing literature

Similar research found that excessive polypharmacy was seen in 24.3% of residents included in the Services and Health for Elderly in Long TERm care (SHELTER) study incorporating 57 European care homes.^8^ Lower prevalence rates in comparison with the present study are likely to be related to differing length of data capture (3 days versus 56 days). Despite differing prevalence rates, the SHELTER study also found that excessive polypharmacy was less common in residents with dementia. A study from Ontario, Canada, analysing a 1-year prescribing cross-section from 2005, found that prescribing of ≥9 drugs was seen in 15.5% of care home residents.^24^ This lower prevalence rate may represent a true difference in practice between Canada and the UK, although the Canadian study used data from 12 years before the present study, when polypharmacy rates were also lower in the general population in Scotland.^7^ A cross-sectional study from Italy showed that antipsychotics were the most commonly prescribed drugs in people with dementia, and proton pump inhibitors in people without dementia.^25^ A large study from France examined prevalence of prescribing of specific potentially inappropriate drugs in care home residents, finding that psychotropic drugs were the most commonly prescribed drug group.^26^ These studies did not systematically examine prescribing by body system, individual and care home predictors associated polypharmacy, or combination prescribing that might put a person at increased risk of ADEs. Studies examining ADEs are less common, and where done tend to examine all older people rather than those living within care homes. One such study of community-dwelling older people from the Republic of Ireland looked at patient-reported ADEs. The study found 74% of the sample population were affected by ADEs, most commonly citing easy bruising, urinary frequency, and ankle swelling; however, it did not examine the prescribing patterns associated with these ADEs.^27^

### Implications for research and practice

The findings show that there is a need to evaluate clinical practice in terms of drug prescribing for care home residents, with careful consideration of the relative benefits and harms in the context of the individual. GPs are responsible for most prescribing for care home residents, with specialist care typically being episodic during acute events.^28^ Primary care prescribing support is required, as well as whole-systems approaches, including all prescribers and technologies, to promote a realistic medicines approach in this population. Prescribers require training and support to deliver safe prescribing and de-prescribing practices, for all residents including those without dementia. This should occur alongside systemic changes, such as technology appraisal, so that safe prescribing is supported from GP IT systems, provision of appropriate national clinical guidance, and adaptation of incentivisation, including the Quality Outcomes Framework (QOF), to account for the complexity of care requirements for care home residents. This involves developing an understanding of real-world, complex treatment regimens in relation to the clinical context, where the risk–benefit balance between prescribing and not prescribing can be examined. Second, the authors' quantification of the magnitude of increased vulnerability to ADEs for residents can be used to inform prescribing practice through identification of the most prevalent potentially inappropriate prescribing of drugs associated with ADEs.

Several areas of uncertainty would benefit from further research. Not all prescribing or polypharmacy is harmful, so it is important to distinguish between appropriate and inappropriate polypharmacy in care home residents. Factors associated with excessive polypharmacy exist at individual and care home levels, and therefore further examination of practices across these levels is needed to identify practices associated with excessive polypharmacy. Considerable unexplained variation was found in prescribing between care homes. Research examining issues, such as care home staff tolerance of behavioural and psychological symptoms of disease (for example, triggering psychotropic prescribing in people with dementia), would be useful.^29^ Further research is needed to identify how best to deliver the optimum balance between burden and benefit.

In conclusion, care home residents have high rates of excessive polypharmacy that is associated with both individual and care home predictors, and there is likely additional variation by region. Potentially inappropriate prescribing of drugs that increase risk of all included ADE categories is common. Further research is required to enable bespoke care home support and provision of medication reviews, including safe prescribing and de-prescribing practices, which facilitate balancing the need for symptomatic relief of symptoms and long-term preventive therapies, against the potential risks of prescribing specific drugs and polypharmacy.
